# Folate-mediated one-carbon metabolism as a potential antifungal target for the sustainable cultivation of microalga *Haematococcus pluvialis*

**DOI:** 10.1186/s13068-023-02353-9

**Published:** 2023-06-17

**Authors:** Hailong Yan, Meng Ding, Juan Lin, Liang Zhao, Danxiang Han, Qiang Hu

**Affiliations:** 1 College of Civil and Transportation Engineering, Shenzhen University, Shenzhen, 518060 China; 2 Center for Microalgal Biotechnology and Biofuels, Institute of Hydrobiology, Chinese Academy of Sciences, Wuhan, 430072 China; 3 Faculty of Synthetic Biology, Shenzhen Institute of Advanced Technology, Chinese Academy of Sciences, Shenzhen, 518055 China; 4 School of Life Sciences, Tsinghua University, Beijing, 100084 China; 5 Poyang Lake Eco-Economy Research Center, Jiujiang University, Jiujiang, 332005 China; 6 Demeter Bio-Tech Co., Ltd, Zhuhai, 519000 China

**Keywords:** Microalgae cultivation, *Haematococcus pluvialis*, *Paraphysoderma sedebokerense*, Fungal contamination, Drug design, One-carbon metabolism, Antifolate

## Abstract

**Background:**

Microalgae are widely considered as multifunctional cell factories that are able to transform the photo-synthetically fixed CO_2_ to numerous high-value compounds, including lipids, carbohydrates, proteins and pigments. However, contamination of the algal mass culture with fungal parasites continues to threaten the production of algal biomass, which dramatically highlights the importance of developing effective measures to control the fungal infection. One viable solution is to identify potential metabolic pathways that are essential for fungal pathogenicity but are not obligate for algal growth, and to use inhibitors targeting such pathways to restrain the infection. However, such targets remain largely unknown, making it challenging to develop effective measures to mitigate the infection in algal mass culture.

**Results:**

In the present study, we conducted RNA-Seq analysis for the fungus *Paraphysoderma sedebokerense*, which can infect the astaxanthin-producing microalga *Haematococcus pluvialis*. It was found that many differentially expressed genes (DEGs) related to folate-mediated one-carbon metabolism (FOCM) were enriched in *P. sedebokerense*, which was assumed to produce metabolites required for the fungal parasitism. To verify this hypothesis, antifolate that hampered FOCM was applied to the culture systems. Results showed that when 20 ppm of the antifolate co-trimoxazole were added, the infection ratio decreased to ~ 10% after 9 days inoculation (for the control, the infection ratio was 100% after 5 days inoculation). Moreover, application of co-trimoxazole to *H. pluvialis* mono-culture showed no obvious differences in the biomass and pigment accumulation compared with the control, suggesting that this is a potentially algae-safe, fungi-targeted treatment.

**Conclusions:**

This study demonstrated that applying antifolate to *H. pluvialis* culturing systems can abolish the infection of the fungus *P. sedebokerense* and the treatment shows no obvious disturbance to the algal culture, suggesting FOCM is a potential target for antifungal drug design in the microalgal mass culture industry.

**Supplementary Information:**

The online version contains supplementary material available at 10.1186/s13068-023-02353-9.

## Background

Microalgae are unicellular or multicellular photosynthetic organisms that live in various habitats and display highly efficient cell growth and photosynthesis [53, 63], which are considered to be promising alternatives for carbon sequestration [9, 36, 63]. Microalgae can accumulate numerous valuable bioproducts that are transformed from photo-synthetically fixed CO_2_ and are therefore applied in many sectors, including food, feed, medicines and biofuels [36]. The industrial-scale cultivation of microalgae to produce high-value products has increased dramatically over the last few decades, but contamination of the mass culturing systems with parasites continues to threaten the sustainable production of algal biomass [6, 37, 64].

The green unicellular microalga *Haematococcus pluvialis* is a freshwater biflagellate that belongs to the class Chlorophyceae, order Volvocales. *H. pluvialis* is well known for its capability in accumulating the valuable carotenoid astaxanthin under stress conditions. The cellular content of astaxanthin accounts for up to 5% of the biomass dry weight of *H. pluvialis*, and thus this organism has become the most sustainable feedstock for the commercial production of astaxanthin [27, 52, 58, 66]. However, the development of the *H. pluvialis* mass culture industry has also been retarded by contamination of the cultures with various microbial pathogens [20, 71]. Among these pathogens, the infection by the parasitic fungus *Paraphysoderma sedebokerense* (Blastocladiomycota) is highly harmful for *H. pluvialis* that causes the most destruction and economic loss to the mass culture of *H. pluvialis* worldwide [15, 26, 31]. Besides *H. pluvialis*, *P. sedebokerense* also infects other economically important microalgae, such as *Chromochloris zofingiensis* and *Scenedesmus dimorphus* [3]. Therefore, uncovering the infection mechanisms of *P. sedebokerense* on microalgal hosts to formulate effective infection controlling methods is important for better production and application of microalgae.

Many efforts have been made to dissect the mechanisms underlying the pathogen–host interactions between *H. pluvialis* and *P. sedebokerense* [4, 25, 39, 40]. Meanwhile, several measures have been deployed to mitigate the threat posed by the pathogen to the natural astaxanthin industry [2, 15, 20, 33]. However, these measures are costly and usually cause reduction in the biomass yield of the *H. pluvialis* [71]. Hence, economically viable, fungi-targeted and algae-safe contamination control measures are desired to improve the sustainability of *H. pluvialis* mass culture.

Identification of the metabolic pathways that are essential for fungal pathogenicity can guide the development of effective control measures [7, 38, 51]. Though most genetic tools of manipulating interesting genes or metabolic pathways are still unavailable for microalgal parasites, utilization of multi-omics approaches can enable the identification of key metabolic pathways [8, 59]. Examples of using such approaches to facilitate understanding the metabolic pathways required for fungal pathogenicity show that by comparative analysis of the genomes of the fungal pathogens and the oleaginous algal host *Nannochloropsis oceanica*, the differences in the key enzymes and genetic structure of the sterol biosynthetic pathway can be identified as targets for commercial fungicides [22]. And by transcriptomic analysis of the interaction processes between the microalga *Graesiella emersonii* and its endoparasite *Amoeboaphelidium protococcarum,* novel insights into robust pathogenicity of the parasite can be gained [14]. Recently, metabolomic analysis has demonstrated that the secondary metabolites, such as 3-hydroxyanthranilic acid and hordenine, can trigger the oxidative burst in *H. pluvialis* cells and can be used by *P. sedebokerense* to facilitate infection [69]. Moreover, based on high-throughput metabolic assay and dual transcriptomic analysis, Lin et al. proved that the metabolic pathways of both carbohydrate-active enzymes (CAZymes) and methionine biosynthesis were essential for the parasitic processes of *P. sedebokerense*, which made it possible to block the infection by using inhibitors targeting CAZymes or methionine biosynthesis [39, 40]. These researches demonstrated the feasibility of omics-based identification of potential metabolic targets that could be used to frustrate fungal infection.

In this study, we conducted RNA-Seq analysis for *P. sedebokerense* upon the infection on its algal host *H. pluvialis*, with the aim to investigate essential metabolic pathways required by the parasite for infection. Many differentially expressed genes (DEGs) related to folate-mediated one-carbon metabolism (FOCM) were found to be enriched in *P. sedebokerense*, and it was assumed that they were essential for the successful parasitization of *H. pluvialis* by *P. sedebokerense*. To verify the hypothesis, the antifolate co-trimoxazole, a combination of sulfamethoxazole and trimethoprim, was applied to the experimental infection systems. The results showed that the infection of *H. pluvialis* by *P. sedebokerense* was restrained under co-trimoxazole treatment. This indicated that FOCM was required for the fungal parasitism, and could potentially be a novel target site for fungicide design in the microalgal mass culture industry.

## Results

### The symptoms and features of *P. sedebokerense* infection in the *H. pluvialis* cell culture

**Fig. 1:**
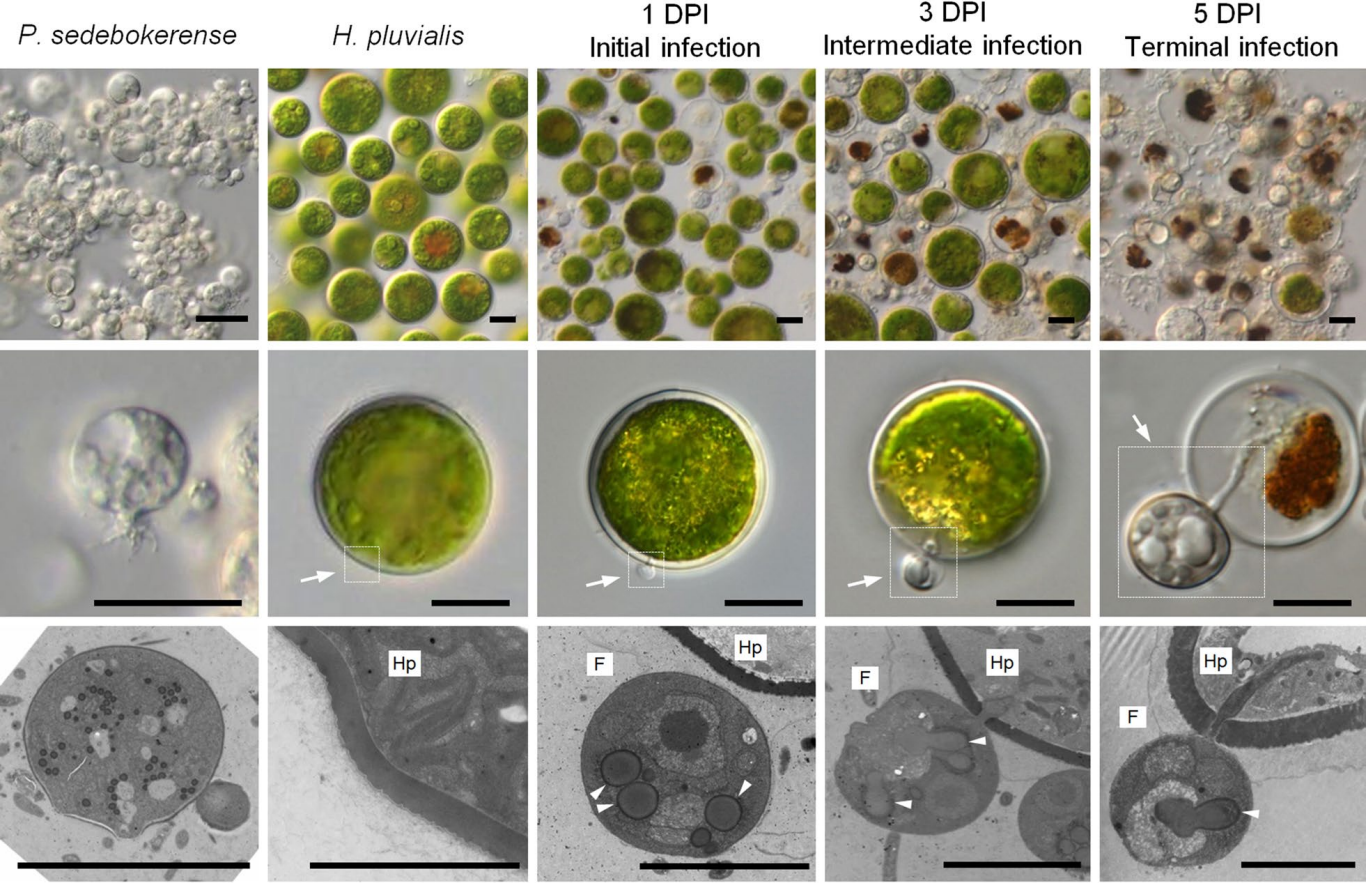
Characterization of the infection features of the parasitic fungus *P. sedebokerense* on its microalgal host *H. pluvialis* at various infection stages. Typical cell features at various infection stages are shown under optical microscope (top and middle) and transmission electron microscope (bottom). White arrows indicate the cell interaction regions of interest; arrowheads indicate lipid droplets inside the fungus cell. *DPI* days post-inoculation. *F* *P. sedebokerense*. *Hp* *H. pluvialis*. Bars = 10 μm

As reported previously, *P. sedebokerense* caused 100% of the infection on the host cells of *H. pluvialis* in 5 days under experimental conditions [69]. Based on the infection ratio and cell developmental features of *P. sedebokerense*, the process was divided into four different stages, consisting of before infection (fungal spores before added to the algal culture), initial infection, intermediate infection and terminal infection (Fig. 1). On the 1st day post-inoculation (DPI), the infection ratio was about 5% and the fungal spores were observed to have accumulated small oil droplets by absorbing nutrients from the host (Fig. 1, arrowheads), which was termed as the “initial infection”. By the 3rd DPI, the number of fungal cells had increased rapidly, the infection ratio had increased to 40–50%, and the volume of the oil droplets in the fungal cells was getting larger. This stage was termed the “intermediate infection” (Fig. 1). By the 5th DPI, the fungal cells had infected nearly 100% of the algal host cells, most of the algal cellular components were digested by the fungus, which made the color of the algal cells brown, and this stage was termed as the “terminal infection” (Fig. 1).

**Fig. 2:**
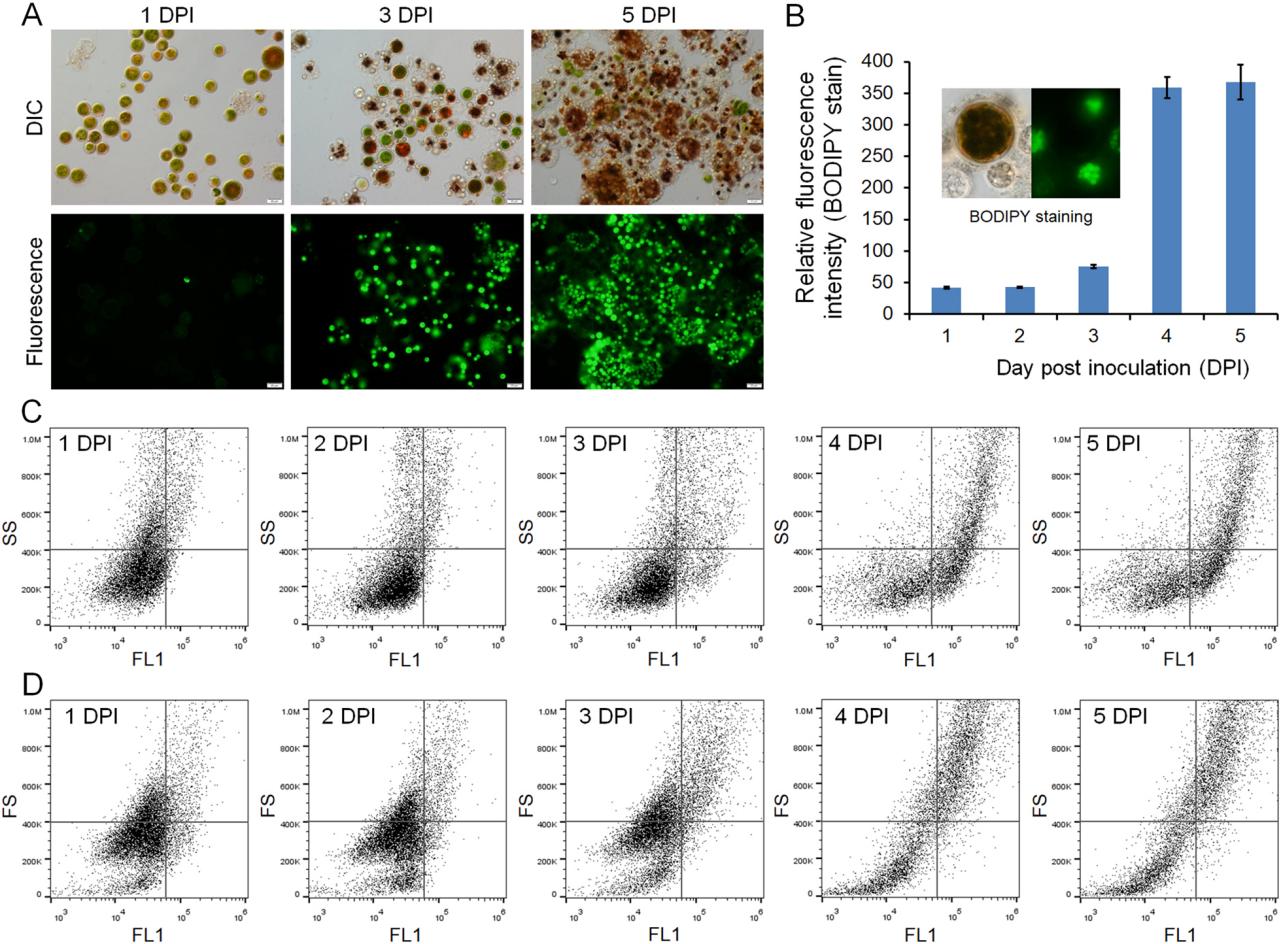
BODIPY staining of algal–fungal co-culture samples collected at various infection stages. **A** Samples observed under optical microscope (top) and fluorescent microscope (bottom). DIC, differential interference contrast. Bars = 20 μm. **B** Quantitative analysis of the fluorescent intensity detected using the cytometer in samples collected at various infection stages. Insert, the BODIPY stained the fungal cells but not the algal cells. **C**, **D** Flow cytometer dot plots showing the correlations between the fluorescence intensity (FL1, x-axis) and side scatter (SS, y-axis, **C**) or forward scatter (FS, y-axis, **D**). DPI, days post-inoculation

In order to further investigate the proliferating pattern of *P. sedebokerense*, a fluorescent dye BODIPY was introduced to stain the lipid droplets of the fungal cells collected at various infection stages. Under the conditions described for the present study, BODIPY stained *P. sedebokerense* cells but not *H. pluvialis* cells (Fig. 2). Therefore, the number of *P. sedebokerense* cells could be qualitatively and quantitatively analyzed by detecting the fluorescence intensity. Fluorescence microscopic observations showed that there were only a few *P. sedebokerense* cells stained with BODIPY and the fluorescence was weak at 1 DPI (Fig. 2A, B). The increased number of stained *P. sedebokerense* cells was observed by 3 DPI, which was in accordance with the fluorescence intensity detected by using the flow cytometry (Fig. 2A, B). The cell number and fluorescence intensity reached the maximum value at 5 DPI (Fig. 2A, B). These results indicated that the proliferation rate of *P. sedebokerense* cells was the highest at 3 DPI during the entire process, suggesting a rapid DNA biosynthesis in *P. sedebokerense*. We speculated that the intermediate infection stage was most important for the fungal parasitism as cell proliferation rate is directly related to new fungal progenies generation, which caused spread of the fungal infection. Correlations between the fluorescence intensity (FL1) and side scatter (SS, provides information about the granularity of the cell sample, representing the number of stained lipid droplets in the cell, Fig. 2C) or forward scatter (FS, allows for the discrimination of cell sample by size, representing the volume of stained lipid droplets in the cell, Fig. 2D) of the flow cytometry, respectively, demonstrated an increase of BODIPY staining in *P. sedebokerense* as the infection developed. Therefore, metabolic pathways such as cell proliferation, organic nutrient assimilation and lipid accumulation could be candidate targets to analysis their requirements for fungal pathogenesis.

### Transcriptomic analysis of *P. sedebokerense* in the infection processes

**Fig. 3:**
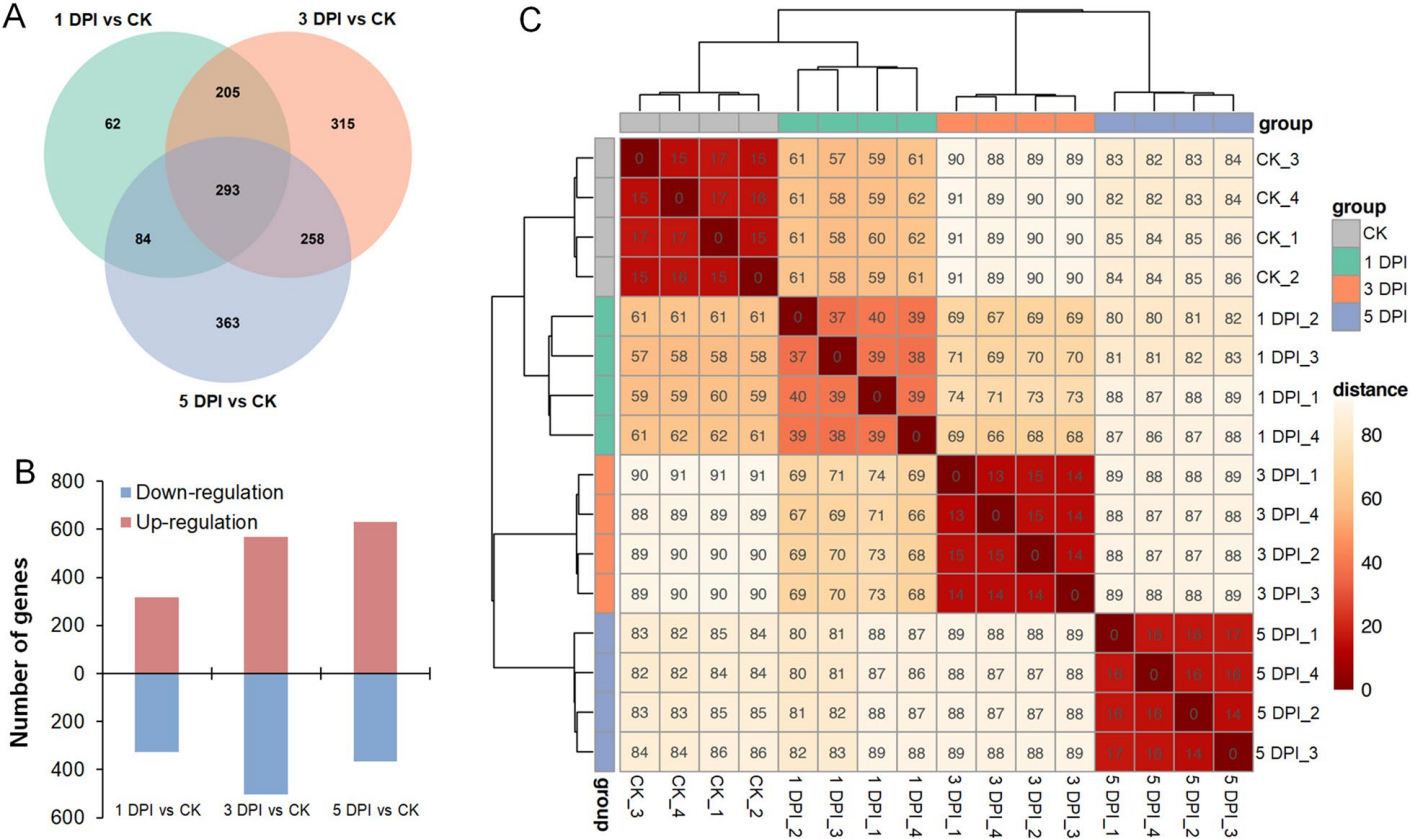
Overview of the transcriptomic results of *P. sedebokerense* upon infection. **A** Venn diagram showing the differentially expressed genes in *P. sedebokerense* at the various infection stages versus the CK. **B** The annotated up- and down- regulated genes in *P. sedebokerense* at the various infection stages versus the CK. **C** The Euclidean distance coefficients of sample-to-sample analysis. *CK* fungal spores before infection; *DPI* days post-inoculation

Transcriptomic analysis of *P. sedebokerense* upon infection was performed on the samples collected at the four stages of infection, including before infection (as the check control, CK), initial infection (1 DPI), intermediate infection (3 DPI) and terminal infection (5 DPI). A total of 77.7 GB of raw data (the RNA-Seq raw data were uploaded to NCBI, with the accession number PRJNA822859) were generated from these samples (4 repeats for each stage). There were 2103, 2059, 2081 and 2082 genes detected in the samples from the CK, 1 DPI, 3 DPI and 5 DPI, respectively. When compared with transcripts of the CK, 644, 1071 and 998 DEGs were characterized in the samples of 1 DPI, 3 DPI and 5 DPI, respectively (Fig. 3A), among which 317, 567 and 631 DEGs were up-regulated, and 327, 504 and 367 DEGs were down-regulated (Fig. 3B). The Euclidean distance coefficients of sample-to-sample analysis among the 16 samples (Fig. 3C) showed that all of the samples were clustered into 4 infection stages with comparatively small distances gathered along the diagonal on the heatmap, which confirmed the accuracy of sampling and the reliability of the RNA-seq analysis.

**Fig. 4:**
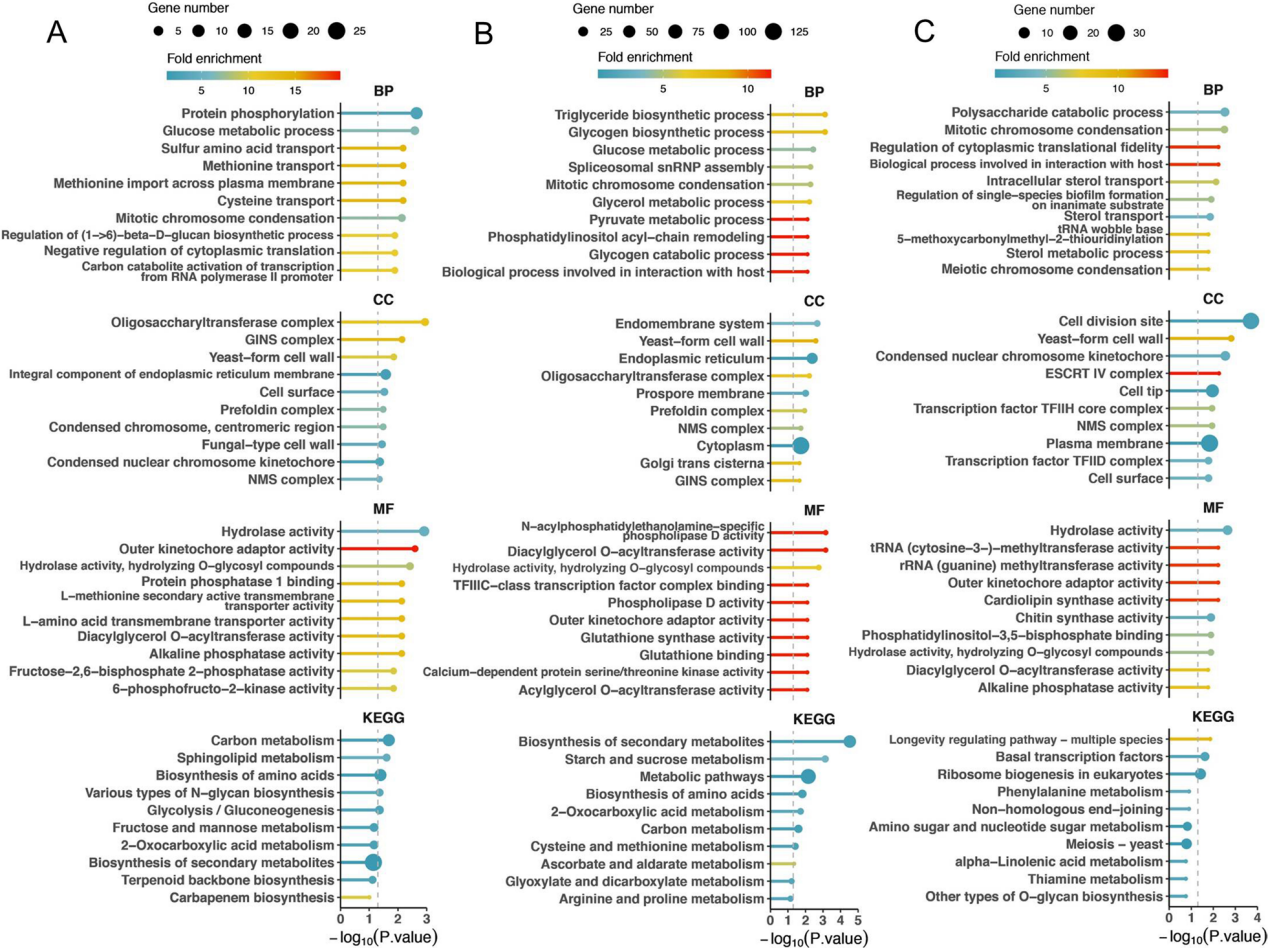
GO analysis and KEGG functional enrichment of differentially expressed genes in *P. sedebokerense* at various stages of infection. The top 10 GO/KEGG terms for the up- and down- regulated differentially expressed genes are shown in **A** 1 DPI vs CK, **B** 3 DPI vs CK, **C** 5 DPI vs CK. Vertical dashed lines correspond to p = 0.05. *BP* biological process; *CC* cellular component; *MF* molecular function; *GO* Gene Ontology; *KEGG* Kyoto Encyclopedia of Genes and Genomes; *CK* fungal spores before infection; *DPI* days post-inoculation

These DEGs might play key roles in the fungal parasitic processes that enable *P. sedebokerense* to successfully colonize the host cells. Therefore, Gene Ontology (GO) and the Kyoto Encyclopedia of Genes and Genomes (KEGG) analyses were carried out on the DEGs to explore the potential functions of the top 10 mostly changed terms (Fig. 4). These analyses indicated that DEGs at 1 DPI were mostly associated with intracellular transportation, translation, hydrolysis activity and carbon metabolism, reflecting the features of the initial invading process of the fungal parasitism (Fig. 4A). At 3 DPI, biosynthetic processes of carbon-mediated metabolism, cell structure formation, amino acid metabolism and secondary metabolite biosynthesis were mostly enriched, indicating fast metabolic changes in the fungal cells (Fig. 4B). At 5 DPI, secondary metabolites, cell division and transcriptional processes were enriched, revealing active secondary growth at this stage (Fig. 4C). We speculated from this overview map that DEGs associated with carbon metabolism could be springboard to analyze the behavior of *P. sedebokerense* upon infection.

### One-carbon metabolism-associated pathways were enriched during the parasitic processes

**Fig. 5:**
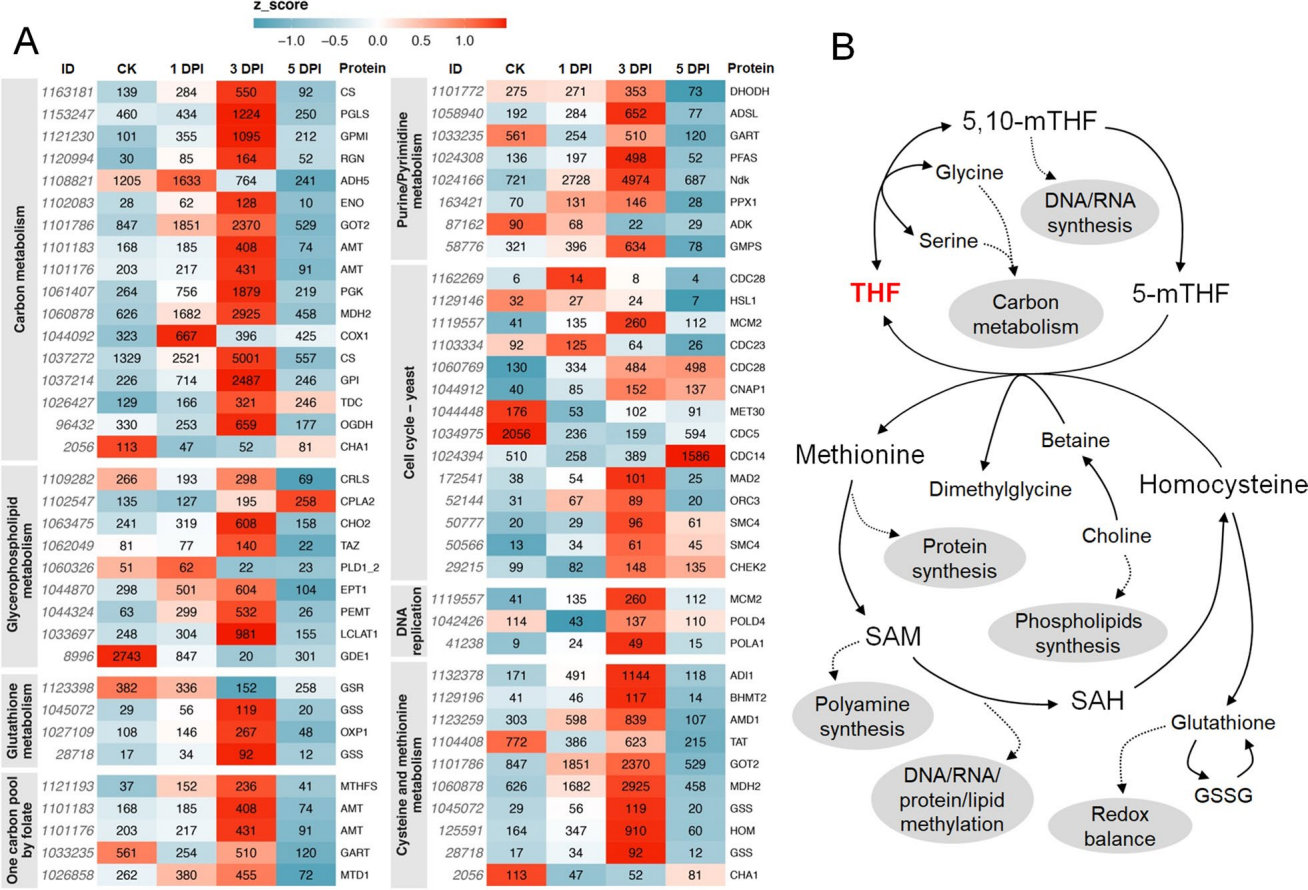
Analysis of the differentially expressed genes associated with the pathway of folate-mediated one-carbon metabolism. **A** Heatmap analysis of the pathways, including carbon metabolism, glycerophospholipid metabolism, one-carbon pool by folate, cell cycle, DNA replication, cysteine and methionine metabolism, purine/pyrimidine metabolism and glutathione metabolism. The full names of the proteins are provided in Additional file 1: Dataset S1. Numbers in the heatmap were mean FPKM value of each gene, color in the box was indicated by z-score. **B** Main processes of folate-mediated one-carbon metabolism. Gray ellipses represent potential metabolism required for the fungal. *THF* tetrahydrofolate; *5-mTHF* 5-methyl-tetrahydrofolate; *5,10-mTHF* 5,10-methylene-tetrahydrofolate; *SAM* S-adenosylmethionine; *SAH* S-adenosylhomocysteine; *GSSG* glutathione (oxidized); *DPI* days post-inoculation

Based on cell staining and microscopic observations, the infection features of *P. sedebokerense* showed obvious differences among the three infection stages, in terms of organic nutrient assimilation, cell division and lipid accumulation, when compared with CK (Figs. 1, 2). Therefore, in our transcriptomic analysis, special interests were paid to pathways of carbon metabolism, lipid metabolism (glycerophospholipid) and DNA biosynthesis (including cell cycle, DNA replication and purine/pyrimidine metabolism). Other pathways possibly associated with the parasitic processes were also analyzed, such as glutathione metabolism, one-carbon pool by folate, and cysteine and methionine metabolism. A heatmap was made by using the metric fragments per kilobase of feature per million mapped reads (FPKM) value of each transcript, and genes associated with the selected pathways were prominent on the heatmap. (Fig. 5A, Additional file 1: Dataset S1). Interestingly, the enriched pathways were related to one-carbon metabolism (Fig. 5B), including the folate cycle, the methionine cycle, and the transsulfuration pathway, collectively referred to as folate-mediated one-carbon metabolism (FOCM) [42]. A number of key DEGs participated in FOCM were hereby selected to analysis their possible roles in *P. sedebokerense*.

In the folate cycle, serine donates one-carbon unit to tetrahydrofolate (THF) to form 5,10-methylene-THF (5, 10-mTHF), which fuels DNA biosynthesis and generates 5-methyl-THF (5-mTHF) (Fig. 5B). A total of 17 DEGs were found to be involved in the biosynthesis of 15 proteins required for carbon metabolism. Among which, glucose-6-phosphate isomerase (GPI), 2,3-bisphosphoglycerate-independent phosphoglycerate mutase (GPMI) and phosphoglycerate kinase (PGK) were three most differentially expressed genes, showing the value of log2 fold-change (log2FC) 3.48, 3.46 and 2.85, respectively, at 3 DPI vs CK (Additional file 1: Dataset S1). Five DEGs were involved in the biosynthesis of 4 proteins required in the pathway of one-carbon pool by folate, among which the 5-formyltetrahydrofolate cyclo-ligase (MTHFS) has a scavenger role as the only enzyme that converts 5-formyl-THF to 5,10-methenyl-THF [46] and showed a value of log2FC 2.69 at 3 DPI vs CK (Additional file 1: Dataset S1). Eight DEGs involving proteins were presented in purine/pyrimidine metabolism, among which the nucleoside diphosphate kinase (Ndk) is a housekeeping enzyme that functions to balance the cellular nucleoside triphosphate pool and was believed to regulate microbial virulence [72], showed a value of log2FC 2.80 at 3 DPI vs CK (Additional file 1: Dataset S1). Three and 14 DEGs involving proteins were presented in pathway of DNA replication and cell cycle–yeast, respectively. Among which, cyclin-dependent kinase (CDC28) was reported to play important role in the control of cell division and modulate transcription in the fungal cells [16], showed a value of log2FC 1.91 at 3 DPI vs CK (Additional file 1: Dataset S1). A majority of these genes showed up-regulated expression patterns upon infection (Fig. 5A, Additional file 1: Dataset S1), especially at the intermediate infection stage (3 DPI), the suspected mostly important infection stage for *P. sedebokerense*, indicating their crucial roles for the fungal pathogenesis.

In the methionine cycle, 5-mTHF relays the one-carbon units to homocysteine to generate methionine, which can be then used for S-adenosylmethionine (SAM) formation (Fig. 5B). SAM acts as a key methyl donor for most methylation modifications, including the methylation of DNA, RNA, lipids and histones, and also participates in polyamine biosynthesis, which has a function in multiple cellular processes including regulation of chromatin structure, transcription and translation, DNA stabilization, cell growth and proliferation [50]. Another component of the methionine cycle, choline, is also a one-carbon unit source after being oxidized to betaine, and is primarily used in lipid biosynthesis [54]. A total of 9 and 10 DEGs encoding proteins that were part of glycerophospholipid metabolism and cysteine and methionine metabolism were discovered in *P. sedebokerense*, respectively. Among these genes, the gene encoding phosphatidylethanolamine/phosphatidyl-N-methylethanolamine N-methyltransferase (PEMT), a transferase enzyme which converts phosphatidylethanolamine to phosphatidylcholine [12], showed a value of log2FC 3.09 at 3 DPI vs CK (Additional file 1: Dataset S1), suggesting up-regulated lipid metabolism in *P. sedebokerense*. Another enzyme, S-adenosylmethionine decarboxylase (AMD1), which is a key enzyme for the synthesis of polyamines [50], showed a value of log2FC 1.49 at 3 DPI vs CK (Additional file 1: Dataset S1), indicating that fungal polyamine biosynthesis was up-regulated in the intermediate infection stage compared with the CK.

In the transsulfuration pathway, glutathione is generated from homocysteine (Fig. 5B), which serves as a critical antioxidant component preserving intracellular redox balance and participating in extensive metabolisms [42, 44]. A total of 4 DEGs associated with glutathione metabolism were identified in *P. sedebokerense*. Among them were two glutathione synthases (GSS) responsible for the synthesis of glutathione, which had log2FC (3 DPI vs CK) values of 2.44 and 2.06, respectively, indicating up-regulation of the glutathione metabolism at the intermediate infection stage versus the CK (Additional file 1: Dataset S1).

Taken together, most of the DEGs related to cell proliferation, organic compound (lipid) accumulation and redox balancing showed up-regulated patterns during the infection process of *P. sedebokerense*. As FOCM supports cell proliferation, lipid accumulation and redox balancing (Fig. 5B), it is reasonable to speculate that FOCM was important for *P. sedebokerense* cell growth and development, and is required for the parasitism.

### Application of antifolate inhibited the pathogenesis of *P. sedebokerense* on *H. pluvialis*

**Fig. 6:**
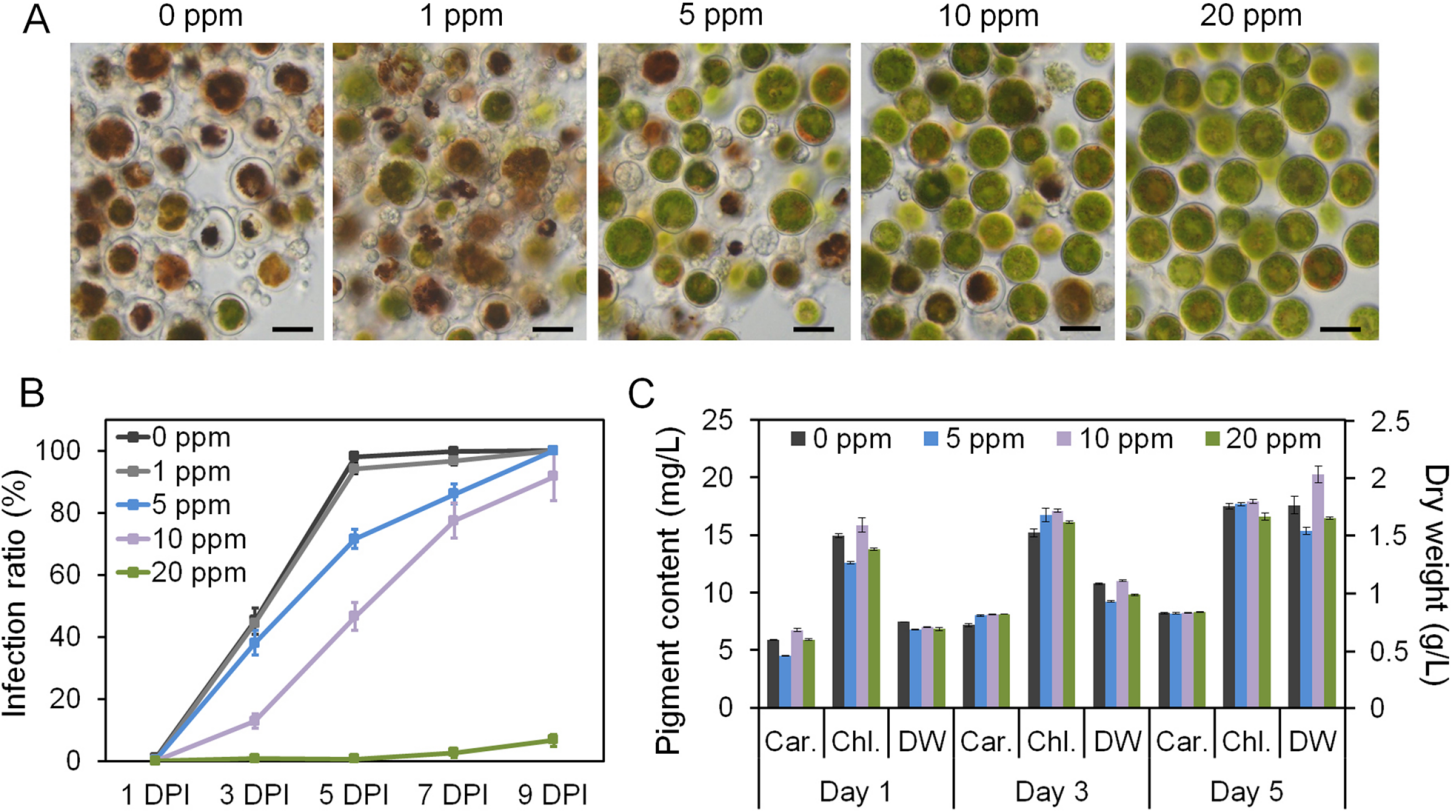
Result of applying co-trimoxazole to the algal–fungal co-culture and the algal mono-culture. **A** Microscopic images showing the inhibitory effect of applying various concentrations of co-trimoxazole to *P. sedebokerense*-infected *H. pluvialis* cultures. The images were taken on the 5th day post-inoculation. **B** The infection ratio in *P. sedebokerense*-infected *H. pluvialis* supplied with various concentrations of co-trimoxazole. **C** Changes in the *H. pluvialis* biomass dry weight (DW) and pigment content, including carotenoids (Car.) and chlorophyll (Chl.), upon co-trimoxazole application. *DPI* days post-inoculation. Bars = 20 μm

The one-carbon metabolism is mediated by folate, and application of antifolates targeting key enzymes in the folate biosynthetic pathway can block the metabolism [21]. Co-trimoxazole is a US Food and Drug Administration-approved drug consisting of two antifolates, trimethoprim and sulfamethoxazole, which are on the list of the World Health Organization essential medicines that have been used as effective antimicrobial agents for over 40 years [34, 70]. The function of these two antifolates is to disorder the cell replication and division cycles in microbes by interfering DNA biosynthesis [17, 23, 35, 56]. Various final concentrations of co-trimoxazole were added to the *P. sedebokerense* and *H. pluvialis* co-culture. The infection ratios were calculated daily to assess the inhibitory effect of co-trimoxazole on the infection. Without the addition of co-trimoxazole, the infection developed rapidly in co-culture reaching an infection ratio of ~ 100% after 5 days inoculation, the algal cellular contents were destroyed by the fungal hypha and the color of the cells turned brown (Fig. 6A, B). With the addition of 1 ppm co-trimoxazole, no significant difference was observed between the treated and untreated co-culture (Fig. 6A, B). Addition of 5 ppm and 10 ppm co-trimoxazole delayed the infection and decreased the infection ratios to 71.5% and 46.7%, respectively, at the 5th DPI (Fig. 6A, B). Elevating the concentration of co-trimoxazole to 20 ppm significantly decreased the infection and the infection ratio was only about 10% after 9 days inoculation (Fig. 6A, B).

The co-trimoxazole was also applied to *H. pluvialis* mono-culture at various final concentrations (0, 5, 10 and 20 ppm). The mono-culture was induced under high light condition to verify the influence of applying co-trimoxazole on algal growth and pigment accumulation (i.e., carotenoids and chlorophyll). When compared with the control (0 ppm), no difference in either biomass dry weight or cell pigments such as carotenoids and chlorophyll was observed in the *H. pluvialis* cell culture exposed to 5, 10 or 20 ppm co-trimoxazole over 5 days of induction (Fig. 6C), indicating that the antifolate would not impose negative impact on the *H. pluvialis* culture.

## Discussion

### Requirement for FOCM for the fungal parasitic process

Tetrahydrofolate and its derivatives are commonly known as folates or B9 vitamins. They can be synthesized de novo in bacteria, fungi and plants and are present in most living cells [24]. Folates are involved in DNA biosynthesis and mediating one-carbon metabolism by transferring one-carbon units from serine to glycine. Folate-mediated one-carbon metabolism fuels the methionine cycle, which supports multiple physiological processes, such as amino acid homeostasis, epigenetic maintenance and redox balance [28, 42]. Numerous studies have demonstrated that FOCM plays essential roles in cell growth and development in higher plants and mammals, particularly in maintaining genome stability and in cell proliferation [24, 42]. It is believed that in proliferating cells, about 40% of nicotinamide adenine dinucleotide phosphate (NADPH), which powers redox defense and reductive biosynthesis, is produced from FOCM [19].

Recently, Yan et al. demonstrated that secondary metabolites produced during *P. sedebokerense* infection on *H. pluvialis* facilitated the infection process via driving oxidative stress to the algal cells [69]. However, the mechanism of *P. sedebokerense* evading off the “oxidative bombing” remained unknown, except for the finding that DEGs involved in oxidoreductase activities were enriched in the pathogenesis of *P. sedebokerense* [39]. In the present study, the up-regulation of genes involved in glutathione biosynthesis offered a possible insight into the strategies used by the fungus to evade oxidative stress upon infection: by up-regulating the DEGs associated with glutathione biosynthesis, *P. sedebokerense* may be able to maintain cellular redox balance under oxidative stress.

**Fig. 7:**
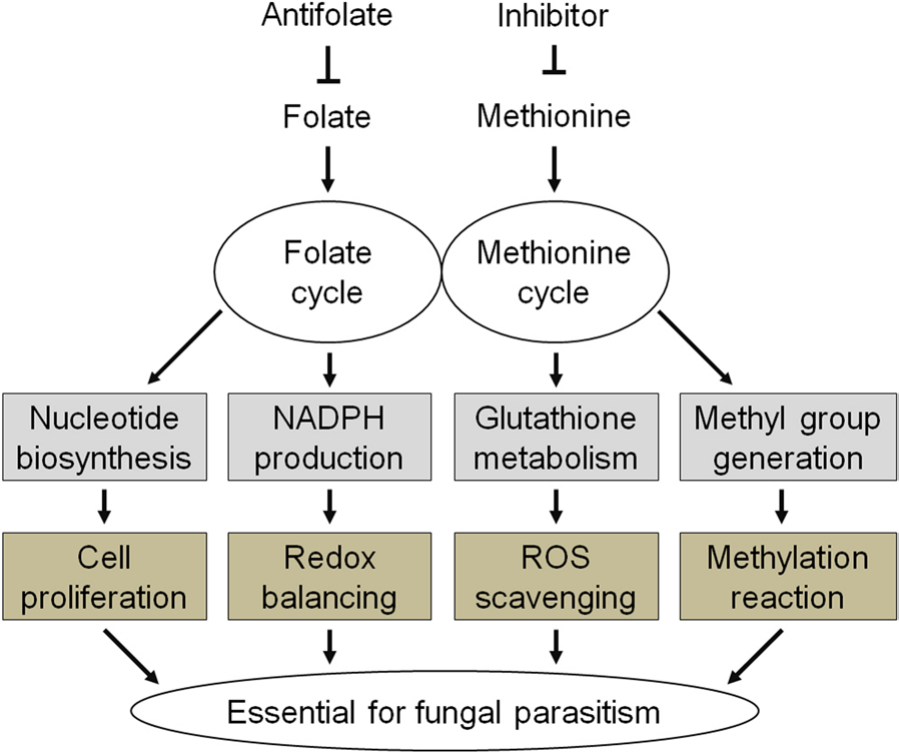
Hypothetical working model showing folate-mediated one-carbon metabolism is required for the parasitism of *P. sedebokerense*. Folate and methionine participate in the folate cycle and the methionine cycle of one-carbon metabolism, respectively. These pathways produce nucleotides, NADPH, glutathione and methyl groups, which are crucial products associated with cell proliferation, redox balancing, ROS scavenging and methylation reaction in the fungus *P. sedebokerense*, allowing it to successfully parasitize its algal host *H. pluvialis*. However, such metabolic processes were arrested by applying antifolates (this study) or inhibitors targeting methionine biosynthesis [40], resulting in restrained infections of *P. sedebokerense* on *H. pluvialis*, collectively suggesting that folate-mediated one-carbon metabolism is required for the parasitism of *P. sedebokerense*

Moreover, Lin et al. showed that application of inhibitors (i.e., pyrimethanil and cyprodinil) targeting methionine biosynthesis, an important part of FOCM, in *P. sedebokerense*-infected algal culture completely abolished the parasitism, suggesting that methionine biosynthesis was essential for the fungal infection [40]. Based on these results, a hypothetical working model is proposed herein to illustrate the possible role of FOCM (Fig. 7). In this model, genes associated with the one-carbon metabolic pathways, including the folate and methionine cycles, are up-regulated in the fungal cells upon infection, and are supposed to result in enhanced biosynthesis of nucleotides, NADPH, glutathione and methyl groups. These products are crucial for cell proliferation, redox balancing, reactive oxygen species (ROS) scavenging and methylation reaction in the fungal cells, which allow the fungus to successfully parasitize the host cells. The nucleotide biosynthesis in the cell proliferation process might ensure rapid propagation of *P. sedebokerense*, which causes a fast spread of the infection. The production of NADPH and glutathione might maintain the redox balance in *P. sedebokerense* and protects the cells from ROS attack. The methylation reaction has been reported to play roles in the growth and virulence of fungal pathogens [29, 30], and is assumed to play the same role in *P. sedebokerense*, possibly controlling the initiation and termination of the pathogenicity through epigenetic modifications and benefiting the pathogen by allowing it to adapt to changing environments upon infection. Though, applications of antifolate or inhibitors targeting methionine biosynthesis were shown to cause restrained infections of *P. sedebokerense* on *H. pluvialis* (the present study and [40]), which leads us to assume that FOCM plays an essential role in the fungal parasitic process, more direct evidences to fully prove the link between folate metabolism and the fungal parasitism are necessary for further underpinning the hypothesis that FOCM is required for the parasitic process.

### Feasibility of applying antifolates to control fungal infection in mass culture of *H. pluvialis*

Microalgae are widely considered as cell factories to synthesize numerous high-value compounds, including lipids, carbohydrates, proteins and pigments [36]. Stress conditions, such as high irradiation, high salinity and nutrient deficiency are introduced to trigger the induction and accumulation of the desired compounds in microalgal cells [53]. Scale-up cultivation of *H. pluvialis* is often realized by a two-stage cultivation method, where cell growth and astaxanthin induction are separated into two steps [18, 41, 73]. The proliferation rate of *H. pluvialis* is fast in the growth stage, but interestingly, fast-dividing *H. pluvialis* cells seem to be unsusceptible to the fungal infection [2]. The fungal contamination frequently happens during the algal induction stage [2, 31], however, the proliferation rate of *H. pluvialis* is significantly low in this stage [32, 58]. Thus, application of antifolates to restrain cell division in the mass culture of *H. pluvialis* is feasible, because blocking of the cell division would cause far more influence on the fungal cells than on the algal cells. In addition, folate is synthesized and stored as poly-gamma-glutamate forms in plant cells and can be hydrolyzed into mono-glutamic forms by the enzyme gamma-glutamyl hydrolase (GGH), which has an important influence on the cellular homeostasis of folate [1, 24, 28]. The GGH encoding gene is present in *H. pluvialis* based on our previous transcriptome data [69], but absent from *P. sedebokerense* (from the present study and also see https://mycocosm.jgi.doe.gov/Parsed1/Parsed1.home.html). This might allow *H. pluvialis* to supply its cellular folate content in the event of folate shortage, and be the possible mechanism by which normal metabolism (i.e., accumulation of biomass and pigments) could be achieved after antifolate application (Fig. 6C).

Admittedly, the risk of using a commixture of antibiotics to protect algal cultures is concerning, as THF is present in most living cells, antifolates might cause inhibition of other organisms in the environment. The chemical co-trimoxazole we used in the study was water soluble, most of the chemical was supposed to remain in the culture supernatant, proper treatment of the supernatant can help lower the risk. Encouragingly, multiple wastewater treatment technologies and media recycling procedures in microalgae cultures have been developed and are well combined with the microalgae mass culture industry, which can remove harmful compounds in the culture efficiently [45, 48, 65]. Therefore, it is possible to apply antifolates to control fungal infection in mass culture of microalgae.

### Screening species-specific antimicrobial agents based on metabolic differences

Fungal pathogens are responsible for extensive losses in both economically important plants and microalgae worldwide, and pose a major threat to global food security [13, 29, 47]. Successful invasion by the fungal parasite on its host requires complicated regulation of pathogenic features during cell growth and developmental processes [5, 11, 57]. Many metabolic pathways, including fungal effector secretion [10], hydrolase production [39], ROS formation [74] and DNA biosynthesis [29], have been found to perform special functions in the fungal invaders upon colonization. Identification of the metabolic differences between microbial pathogens and their hosts, as well as the design of species-specific antimicrobial agents targeting the differential metabolic pathways, therefore represent the most promising fields of research and are strongly supported by recent developments in biological methodology and system biology that allow a better understanding of pathogenic mechanisms at various levels [8, 22, 59]. In this study, FOCM was characterized and differences in requirements between *P. sedebokerense* and *H. pluvialis* were shown to occur during the infection process, suggesting that FOCM could potentially be a target site for the development of fungal-specific inhibitors.

The next generation of microalgal mass culture will be built on the foundation of extensive application of big data analysis and active intervention, which will allow not only the optimal process conditions to be determined for maximal production of microalgal biomass and bioproducts [49, 62], but also tailored contamination control strategies to be formulated to guarantee the quality of the products [22, 71]. The present work offers a case study of the discovery of a new antimicrobial cure from an old drug, based on the metabolic features of the pathogen, and as such will help inform further developing the species-specific contamination control approaches for sustainable microalgal cultivation.

## Conclusion

In the present study, RNA-Seq analysis of the DEGs in *P. sedebokerense* at various infection stages revealed enriched metabolic pathways in FOCM, which was assumed to be critical for both cell proliferation processes and redox balancing in the fungal cells. By applying antifolate targeting folate biosynthesis, the parasitic process was inhibited, suggesting that folate plays an important role during the fungal parasitism. We draw a conclusion from these results, together with other evidence of a similar inhibition of infection by addition of inhibitors targeting methionine biosynthesis, that FOCM is essential for the parasitic process of *P. sedebokerense*. This evidence helps advance understanding of the mechanisms underlying the interaction between fungal parasites and microalgal hosts, and provides a new consideration for antimicrobial drug design in the microalgal mass culture industry.

## Materials and methods

### Biological material cultivation conditions

*H. pluvialis* strain K-0084 was acquired from the Scandinavian Culture Center for Algae and Protozoa (SCCAP) at the University of Copenhagen, Denmark. The alga was cultured in BG11 growth medium, which contained (per liter): KNO_3_, 1500 mg; K_2_HPO_4_, 40 mg; MgSO_4_·7H_2_O, 75 mg; CaCl_2_·2H_2_O, 36 mg; citric acid, 6 mg; ferric-ammonium citrate, 6 mg; EDTA-Na_2_, 1 mg; Na_2_CO_3_, 20 mg; H_3_BO_3_, 2.86 mg; MnCl_2_·4H_2_O 1.81 mg; ZnSO_4_·7H_2_O, 0.22 mg; CuSO_4_·5H_2_O, 0.08 mg; Na_2_MoO_4_·2H_2_O, 0.39 mg and Co(NO_3_)_2_·6H_2_O, 0.05 mg [60]. The algal cells were cultured in 800 mL column photobioreactors at 21–23 °C under continuous illumination (~ 20 μmol m^−2^ s^−1^) with aeration of 2% (*v/v*) CO_2_. The fungal parasite used in this study, *P. sedebokerense* (GenBank accession number MN203631), had been isolated in the previous study [39] and was cultured in the enriched chytrid growth medium, which contained the modified BG11 medium (inorganic carbon and nitrogen were depleted) supplied with 5 g L^−1^ glucose, 2.5 g L^−1^ peptone and 1.25 g L^−1^ yeast extract [31]. An initial fungal cell density of OD_600_ = 0.03 was inoculated into the 100 mL enriched chytrid growth medium in 250 mL flasks and cultured on a shaker incubator at 30 °C, 150 rpm under continuous illumination (~ 10 μmol m^−2^ s^−1^). Cell subculturing was conducted weekly by 5% (*v/v*) of the fungal cells to the fresh enriched chytrid growth medium.

### Infection assay

Once the algal cells in the 800-mL column photobioreactors reached the exponential phase, most of the cells became green cyst that could be infected by *P. sedebokerense*. The algal cells were centrifuged at 700*g* for 3 min and the pellet was re-suspended in 100 mL BG11 medium at the cell density of 4 × 10^5^ mL^−1^ and was transferred into the 250-mL flasks. These algal cell cultures were then inoculated with *P. sedebokerense.* The fungal inoculum was prepared using axenic fungal cell cultures grown for 5 days, from which 1 mL of the fungal suspension was pelleted through centrifugation and was re-suspended in BG11 medium, which was then used as the inoculum. The algal–fungal mixture was incubated on an orbital shaker set at the speed of 150 rpm at 30 °C under continuous illumination (~ 20 μmol m^−2^ s^−1^). Daily sampling was performed to monitor the fungal infection process under microscope (Olympus, BX53 with a DP70 CCD camera). The infection ratios were calculated by using the equation described by Gutman [26]. Over 1000 algal cells were examined with three replicates to calculate the infection ratio and the quantitative data were presented as mean ± S.D.

### Transmission electron microscopy and BODIPY staining

Samples collected from each infection stage were fixed in 2.5% (*v/v*) glutaraldehyde at 4 °C overnight, and were washed with 0.1 M phosphate buffer saline (PBS), post-fixed in 1% (*w/v*) osmic acid for 2 h, washed again with 0.1 M PBS, dehydrated sequentially with ethanol gradient, and infiltrated with epoxy. The samples were cut into 60 nm thin sections and were observed with a 200 kV transmission electron microscope (Tecnai G2 20 TWIN, 0.24 nm/200 kV, FEI Company, USA). The fluorescent dye BODIPY (Molecular Probes, USA) was used to stain the cells for detection of intracellular lipid droplets [55]. After BODIPY staining (final concentration 50 μM) for 10 min in the dark at room temperature (under such conditions, *H. pluvialis* cells were not stained because of their thick cell wall), the samples were observed under the fluorescent microscope and the fluorescence intensity of the stained fungal cells was measured with the flow cytometer (Beckman Coulter, Inc. FC-500, USA). The excitation/emission wavelength was 488/525 nm. Three replicates were included to calculate the fluorescence intensity, and the quantitative data were presented as mean ± S.D.

### Measurement of cell growth and pigments

The biomass dry weight of the antifolate-treated algal cell cultures was measured on a daily basis. 10 mL of the cell culture were collected and filtered through the Whatman GF-C glass microfiber filters. The filters were then washed with 0.5 M ammonium bicarbonate to remove salts and dried in a 105 °C oven for 24 h. Four replications were conducted for each measurement. To extract pigments from the samples, 1-mL aliquots of the inoculated cell culture were pelleted through centrifugation and re-suspended in dimethyl sulfoxide (DMSO). A quarter volume of 0.4–0.6 mm glass beads were added to the cell suspension. The suspensions were treated with a cell disrupter (Mini-BeadBeater-24, USA) for several times under dim light (less than 0.02 μmol m^−2^ s^−1^) until the cell pellet turned colorless. The DMSO extracted pigments were collected by centrifuging at 3000*g* for 1 min. The concentrations of chlorophylls and carotenoids were quantified by using a DR6000 UV–vis spectrophotometer (HACH company, USA) [67], and the quantitative data were presented as mean ± S.D.

### RNA extraction and RNA-Seq analysis

Samples of 1 mL algal–fungal co-culture and fungal cell suspensions before infection were harvested by centrifuging at 1200*g* for 5 min. The cell pellets were immediately frozen in liquid nitrogen and stored at − 80 °C until RNA extraction. Four replicates were prepared for each sample. The total RNA of the samples was extracted with Trizol reagent (Invitrogen, USA) according to the manufacturer’s instructions. The RNA was quantified using a Nanodrop 2000 spectrophotometer (Thermo Fisher Scientific, USA), and the RNA integrity was assessed using an Agilent 2100 system and RNA Nano 6000 Assay kit (Agilent Technologies, USA). Qualified RNA was reverse transcribed into double-stranded cDNA and was sequenced for 2 × 150-bp runs (paired-end) using a Hiseq 2000 sequencing system (Illumina, USA), this being performed at Bioacme Biological Technologies Corporation (Wuhan, China). The transcriptomes of *P. sedebokerense* was constructed with the reference genome from the Joint Genome Institute (https://jgi.doe.gov/). Differentially expressed genes (DEGs) were identified by comparing the infected samples with the CK samples with the DESeq2 software package after a routine of robust quality checks [43]. The genes with a p-value of ≤ 0.05 and log2 fold-change absolute value of > 1 were considered as DEGs. The GO and KEGG enrichment analysis were conducted by using the clusterProfiler package in R [68], with the p-value of enriched GO terms, including biological process (BP), cellular component (CC) and molecular function (MF), and KEGG pathways calculated using a hypergeometric distribution [61]. The top 10 ranked categories from the analyses were identified to globally determine the functional roles and related pathways of the DEGs. To evaluate the similarity of the samples in each infection stage, hierarchical clustering analysis that visualized the Euclidean distance between all the samples was calculated from the regularized-logarithm transformation (rlog) using the “pheatmap” package in R. Notably, heatmap showing the expressions of significantly expressed genes from the pathways of interest was generated by the R package “ggplot2” using the metric FPKM, and was colored by the z-score that was computed from the average FPKM of samples in each stage by subtracting the value of the average FPKM in the four stages then dividing by the standard deviation.

### Application of antifolates to *H. pluvialis* cell culture

The antifolate additive used in this study was co-trimoxazole (a combination of sulfamethoxazole and trimethoprim). The co-trimoxazole was dissolved in BG11 medium to give a concentration of 1 × 10^3^ ppm as stock solution. The stock solution was added to the 100 mL algal–fungal co-cultures grown in 250 mL flasks to give final concentrations of co-trimoxazole of 1, 5, 10 and 20 ppm, respectively. The co-cultures were incubated on an orbital shaker set at a speed of 150 rpm, 30 °C, under a light intensity of ~ 20 μmol m^−2^ s^−1^. Daily sampling for microscopic observations was performed to calculate the fungal infection ratios. Various final concentrations of co-trimoxazole were also added to *H. pluvialis* mono-culture. The initial cell density of the algal mono-culture was about 6 × 10^5^ mL^−1^ and the cultures were induced in 800 mL column photobioreactors under high light illumination (~ 150 μmol m^−2^ s^−1^). Samples were collected at 1, 3 and 5 days of the induction, the biomass dry weight and pigment content (including carotenoids and chlorophyll) of the algal cells were measured to verify the influence of co-trimoxazole on *H. pluvialis*.

## Supplementary Information

**Additional file 1: Dataset S1.** Raw data of the FPKM value genes analyzed in heatmap.

## Data Availability

Raw data of the transcriptome analyzed in this work are available at NCBI Sequence Read Archive (https://www.ncbi.nlm.nih.gov/sra) with the accession number PRJNA822859.
